# The Role of Yield Components in Pea (*Pisum sativum* L.) Yield Adaptability

**DOI:** 10.3390/plants15192903

**Published:** 2026-09-23

**Authors:** Tihomir Čupić, Ivana Plavšin, Branimir Tokić, Sonja Petrović, Marijana Tucak, Goran Krizmanić

**Affiliations:** 1 Department of Forage Crops Breeding and Genetics, Agricultural Institute Osijek, Južno Predgrade 17, 31000 Osijek, Croatia; tihomir.cupic@poljinos.hr (T.Č.); marijana.tucak@poljinos.hr (M.T.); goran.krizmanic@poljinos.hr (G.K.); 2 Department of Cereal Breeding and Genetics, Agricultural Institute Osijek, Južno Predgrade 17, 31000 Osijek, Croatia; ivana.plavsin@gmail.com; 3 Faculty of Agrobiotechnical Sciences Osijek, Josip Juraj Strossmayer University of Osijek, Vladimira Preloga 1, 31000 Osijek, Croatia; sonja.petrovic@fazos.hr

**Keywords:** phenotypic plasticity, yield stability, yield components, seed productivity, genotype × year interaction, *Pisum sativum* L.

## Abstract

Yield variation across environments is closely related to the response of individual yield components to environmental conditions. In this study, nine pea (*Pisum sativum* L.) breeding lines were evaluated over four growing seasons at the site where they were selected. Seed productivity per plant, number of pods per plant, number of seeds per pod, and thousand-seed weight were measured at the individual plant level. Linear mixed-effects models were used to estimate the effects of genotype, year, and genotype × year interaction on seed productivity per plant and to assess whether the relationships between yield components and this trait changed between growing seasons. A significant genotype × year interaction indicated that genotypes responded differently across growing seasons. Pod number showed the strongest association with seed productivity per plant and the largest standardized effect. In contrast, the interaction between year and thousand-seed weight was not significant, indicating a relatively consistent relationship with seed productivity across growing seasons. These results show that the relationships between individual yield components and seed productivity changed across growing seasons. Identifying the importance of yield components under varying environmental conditions provides useful phenotypic information for understanding yield stability and adaptive responses to interannual environmental variation and may contribute to breeding for improved performance under growing conditions.

## 1. Introduction

Maintaining stable seed yield under changing environmental conditions remains a major challenge in crop production. Seed yield is strongly influenced by environmental conditions, while genotype responses may differ among growing seasons and production environments. Genotype × environment interactions therefore account for an important part of yield variation in many crops [1,2]. Breeding programs aim to develop genotypes for defined target environments, and the choice of the breeding environment is closely related to the intended production environment [3]. Adaptation can be considered at different scales, from specific adaptation to particular environments to wider adaptation across environments [4]. Parametric, nonparametric and multivariate approaches are often used to assess genotype performance and stability in multi-environment experiments. However, these methods mainly describe genotype responses and provide less information about the biological processes involved in yield formation [5,6]. In pea (*Pisum sativum* L.), seed yield is determined by closely related reproductive components, including pod number, seed number per pod and seed weight, whose relative contributions may vary among genotypes and growing conditions [7,8,9]. These components may also differ in their responsiveness to environmental variation, as shown by differences in the expression of pea yield-related traits across environments [10]. Seed numbers are often more responsive to resource availability, while seed weight may vary less across environments [11,12]. Environmental conditions during flowering and seed development may affect the contribution of individual reproductive traits to final seed yield [13,14,15].

In pea production, yield reductions are often associated with poor pod set, particularly under high temperatures or limited rainfall during flowering [14,16]. Seed weights usually vary less between years than pod number, indicating different levels of environmental sensitivity among yield components [12,15,17]. The response of individual yield components is therefore not uniform. Reduced seed number may in some cases be partly compensated by increased seed weight, depending on resource availability and developmental responses [12].

The main components of pea yield, including pod number, seed number per pod and thousand-seed weight, are under complex genetic control and are closely interconnected. Their relative contributions to seed yield may change across environments owing to compensatory relationships among traits [8,9]. Thus, a decrease in one yield component may be partially compensated by an increase in another component, such as higher seed weight when seed number is reduced [12]. Changes in yield components in response to environmental variation reflect phenotypic plasticity and may contribute to dynamic yield stability when changes in one component are partly compensated by changes in another [11,18]. Such relationships are particularly relevant for understanding how reproductive traits contribute to yield under variable growing conditions. Phenotypic evaluation across contrasting environments can therefore help identify reproductive traits associated with yield responses to environmental variation and provide a basis for future breeding strategies [19,20].

High average yield is not always associated with stable performance across different environments [6]. Differences in yield performance may be related to variation in reproductive development and genotype-specific responses to environmental conditions [20,21]. Multi-environment experiments are commonly used to evaluate genotype performance across years or locations. Linear mixed-effects models can simultaneously evaluate genotype, year, and interaction effects within a unified analytical framework [6,22,23]. In pea, such approaches are particularly relevant because reproductive development is sensitive to environmental conditions during flowering and early seed development. Yield components do not respond equally across environments, and the magnitude of these responses may differ among genotypes [9]. Previous studies in pea have investigated the stability of yield and yield components across years and environments. These studies have found significant genotype × environment interactions as well as differences in the stability of seed yield and its components [1,17,24,25]. Other authors have investigated the relationships between yield components using correlation and path analysis and have found differences in the relationships among components between genotypes and in their contribution to seed yield [8]. However, less attention has been paid to changes in the relationships between individual yield components and seed productivity across growing seasons and how these changes are related to interannual environmental variation. This study therefore focuses on the year-dependent relationships between individual yield components and seed productivity, rather than only on changes in the magnitude of individual yield components across environments.

Therefore, the main objective of this study was to examine how genotype, growing season, and genotype × year interaction affect seed productivity per plant and how the relationships between individual yield components and seed productivity change across growing seasons. The secondary objectives were to (i) quantify the relative associations of pod number, number of seeds per pod, and thousand-seed weight with seed productivity per plant, (ii) determine whether these relationships vary among growing seasons, and (iii) identify yield components showing stronger environmental responsiveness or more consistent relationships with seed productivity across growing seasons.

Based on previous findings, we expected that pea genotypes would differ in their seed productivity responses across growing seasons and that the main yield components would be associated with seed productivity per plant. We also expected that the strength of these relationships would vary among growing seasons, reflecting differences in the phenotypic plasticity of individual yield components under interannual environmental variation. By comparing yield components across growing seasons, we gained insight into their interrelationships, how individual yield components respond to changing environmental conditions, and how these responses are related to yield stability. These differences may help identify yield components that are more responsive to environmental variation or more consistent across growing seasons, which could be useful for breeding under changing growing conditions.

## 2. Results

### 2.1. Environmental Variation in Yield and Yield Components

Descriptive statistical analysis revealed clear differences between growing seasons in seed productivity and yield components (Table 1). The highest seed productivity per plant was recorded in 2020, followed by generally lower values in the subsequent experimental growing seasons, with the lowest seed productivity observed in 2024. Seed productivity per plant variability was greatest during the last two experimental growing seasons (2022 and 2024). Among the yield components, pod number showed considerable differences among growing seasons, with the lowest mean observed in 2024. Thousand-seed weight remained relatively similar during the first three years but decreased in 2024, while the number of seeds per pod remained relatively similar during 2021 and 2022 and increased in 2024.

Average seed productivity differed among genotypes and growing seasons, indicating the presence of genotype × year interaction (Figure 1). Genotype rankings changed across growing seasons, reflecting differential genotype responses to year-to-year variation.

### 2.2. Genotype and Environmental Effects on Yield Variation

Seed productivity was analyzed using a linear mixed model including year, genotype, and the genotype × year interaction as fixed effects, while PID was included as a random intercept (Table 2). The factor year (Y) had a highly significant effect on pea seed productivity (F = 15.19, *p* < 0.001), while in contrast, the effect of genotype (G) was not statistically significant (*p* = 0.066). The genotype × year (Y × G) interaction was highly significant (F = 2.57, *p* = 0.001), with different genotype responses among years, as shown in Figure 1. Model I explained 19.0% of the total productivity variation with fixed effects (marginal R^2^), while plot-level random effects increased the explanation of the total variance to 25.0% (conditional R^2^). The results indicate that year-to-year variation was strongly associated with seed productivity, while genotype performance differed among growing seasons because of the significant genotype × year interaction.

### 2.3. Relationships Between Yield Components and Seed Productivity

Model II incorporated yield components together with their interactions with year to evaluate both the contribution of reproductive traits to seed productivity and whether these contributions varied among growing seasons (Table 3). Model II substantially improved model performance compared with Model I, as indicated by the increase in explained variance. The marginal R^2^ increased from 0.190 in Model I to 0.820 in Model II, indicating that a large proportion of yield variation was explained through variation in yield-component traits.

All estimated yield components showed a highly significant association with seed productivity (*p* < 0.001). The number of pods per plant showed the strongest statistical association with seed productivity (F = 1617.35), followed by the number of seeds per pod (F = 468.46) and thousand-seed weight (F = 378.81). Significant interactions between year and pod number, as well as between year and seeds per pod, indicated that the contribution of these traits varied among growing seasons. In contrast, the interaction between year and thousand-seed weight was not significant (*p* = 0.896), indicating that the relationship between thousand-seed weight and seed productivity did not differ significantly among years.

The effect of year remained highly significant after including yield components, although its magnitude was reduced compared with Model I. This indicates that part of the year-related variation in seed productivity was associated with variation in yield components. In contrast to Model I, the genotype effect was significant in Model II (*p* = 0.001) after including yield components and their interactions with year, indicating that genotype differences in seed productivity were detected after accounting for these effects.

To further assess the relative importance of individual yield components, the predictors included in Model II were standardized using a z-score transformation (Table 4). This allowed for comparisons between traits measured on different numerical scales. The standardized model retained a very high level of model explanatory power, with marginal and conditional R^2^ values of 0.820 and 0.825, respectively. The trait number of pods per plant had the largest standardized effect on seed productivity (β = 2.743), while thousand-seed weight (β = 1.356) and number of seeds per pod (β = 1.255) had a smaller effect. These results indicate that number of pods per plant was the strongest predictor of seed productivity across growing seasons.

The relationship between the number of pods per plant and seed productivity is shown in Figure 2A, while Figure 2B shows the relationship between seed productivity and the number of seeds per pod across different years. The regression lines for seed productivity against both yield components differed among growing seasons. The number of pods per plant had slopes of 0.614, 0.456, 0.760 and 0.774 in 2020, 2021, 2022 and 2024, while the R^2^ values were 0.386, 0.258, 0.705 and 0.506. The intercepts ranged from 0.372 in 2022 to 2.901 in 2021. The number of seeds per pod showed slightly different values, with slopes by year of 1.118, 0.870, 0.700 and 0.651 and R^2^ values of 0.147, 0.206, 0.034 and 0.057, while the intercepts ranged from 2.772 in 2024 to 4.428 in 2022. No statistically significant differences were found for the interaction between thousand-seed weight and year.

## 3. Discussion

Weather conditions varied among the experimental growing seasons and were associated with differences in seed yield and the expression of yield components in pea. The reduction in seed productivity and the greater variability observed in the later growing seasons suggest that reproductive development was sensitive to environmental variation. Flowering, pod set and seed filling are particularly sensitive stages because temperature and water availability can affect reproductive development and assimilate allocation. Similar responses have been described by [14], who reported reduced pollen germination and pod development under high-temperature stress. Environmental stress during reproductive growth can also alter assimilate partitioning and increase yield variability among environments [26]. These changes can affect the relative contribution of individual yield components to seed productivity.

Among the yield components analyzed, pod number showed the strongest association with seed productivity. Previous studies on peas have shown that pod number is strongly associated with seed yield and that reproductive traits respond to environmental conditions [8,9,15]. Thousand-seed weight is generally less sensitive to environmental conditions than pod number. It may remain relatively stable when pod number or pod size decreases [15,17]. In our study, the relationship between pod number and seed productivity significantly changed among growing seasons, whereas no significant year × thousand-seed weight interaction was detected.

The genotype effect was relatively small in this study, which may be explained by the common breeding origin and similar selection objectives of the evaluated lines. The evaluated lines originated from the same breeding cycle and were selected under similar breeding objectives focused on seed yield within the target breeding environment where the field experiment was conducted. Despite their similar overall productivity, the significant genotype × year interaction showed that the lines responded differently among growing seasons. This indicates that similar levels of seed productivity can be achieved through different responses to environmental variation. When yield components were included in Model II, the genotype effect became significant, suggesting that part of the genotype differences was expressed through variation in yield components.

The results obtained are consistent with the concept of dynamic stability, in which genotype performance depends on responses to environmental variations [22,27,28,29]. Genotype rankings changed over the growing seasons, indicating that the genotypes tested responded differently from year to year. The conclusions based on the present results are consistent with previous findings and support the importance of phenotypic plasticity for maintaining yield under changing environmental conditions [11,18].

A substantial increase in explained variance was observed after incorporating yield components and their interactions with year. The results indicate that seed productivity variation in pea was strongly associated with variation in individual reproductive traits, in addition to genotype and year effects. This finding indicates that variation in final yield is strongly associated with several yield components rather than with a single trait, consistent with previous findings in pea and other crops [1,9].

Among the evaluated yield components, pod number showed the strongest association with seed productivity. Similar observations have been reported in pea and other legumes, where pod number was identified as one of the yield components most closely associated with final seed yield [8,9,17]. The significant interactions between year and both pod number and seeds per pod indicate that the relationships between these reproductive traits and seed productivity varied among growing seasons. The regression analysis further showed that the relationships of pod number and seed number per pod with seed productivity were not constant among growing seasons. In contrast, the relationship involving thousand-seed weight remained more consistent across years. This variation reflects the plastic response of reproductive traits to changing environmental conditions. Similar responses of reproductive traits under drought and heat stress have been reported in pea and other legumes [14,15,26,30].

The different responses of yield components indicate that yield stability does not necessarily require stability of each component. Yield components are formed at different stages of reproductive development. Pod number is largely determined during flowering and early pod development, whereas final seed number and seed filling are determined later. A reduction in pod number may therefore be partly offset by subsequent development of the remaining reproductive structures. Changes in the number of pods and seeds per pod across growing seasons, together with the relatively stable thousand-seed weight, indicate that yield formation depends on the contribution of different reproductive components under changing environmental conditions. Such compensatory relationships between yield components have been described in crops, where changes in one component can be partially compensated by changes in another [12,31].

In contrast to the number of pods per plant and the number of seeds per pod, the interaction effect for thousand-seed weight was not significant. Similar responses have been reported in pea, where environmental stress during reproductive development can affect seed filling and seed weight [15,26]. The relatively stable response of thousand-seed weight, despite changes in the other yield components, further suggests that individual components may contribute differently to yield formation across growing seasons. Previous studies have shown that environmental stress during flowering and seed filling may alter the contribution of reproductive traits to final seed yield and lead to substantial differences in genotype responses across environments [14,15,26].

Traditional analytical tools, such as AMMI and other stability analyses, are valuable tools for identifying stable and widely adapted genotypes across different environmental conditions [5]. These approaches mainly describe genotype responses and provide less direct insight into the biological factors underlying yield stability. In contrast, the linear mixed-effects modelling approach used in the present study enabled simultaneous evaluation of the effects of individual yield components and their interactions with year, providing additional insight into phenotypic plasticity and the relative importance of reproductive traits for seed productivity.

The present results indicate that analyses of yield components can complement conventional stability approaches by providing information on how individual components contribute to seed productivity under different growing conditions. For breeding purposes, the results suggest that plants should be selected for consistency of pod number across growing conditions rather than for maximum pod number in a single favorable season. Since pod number showed the strongest association with seed productivity but also the greatest environmental responsiveness, stability of pod formation may be a more useful selection target than its maximum expression. The different responses of the reproductive components further suggest that stable pod formation should be considered together with the capacity of the plant to maintain seed set and seed filling under variable conditions, although this compensation was not directly tested in the present study. Thousand-seed weight showed a comparatively stable relationship with seed productivity and could therefore be used as a more stable selection trait. However, this does not necessarily imply that genotypes with the largest seeds should be preferred. Although the relationship between seed size and compensatory responses among yield components was not directly examined in the present study, our long-term breeding experience suggests that genotypes with moderate and relatively stable thousand-seed weight may be more suitable for maintaining stable seed productivity than genotypes selected primarily for very high thousand-seed weight.

The results of this study should be considered within the target breeding environment in which the lines were developed and selected. Multienvironment evaluation has also been used in pea breeding to identify breeding lines adapted to specific production conditions [32]. Previous modelling work in pea has further shown that identifying traits associated with adaptation to future climatic conditions can support the definition of breeding ideotypes [33]. Therefore, evaluation in additional target environments would be needed to determine whether the observed relationships are consistent across different production environments.

The relationships between environmental variability, yield component plasticity, genotype responses and yield stability are summarized in the conceptual framework presented in Figure 3.

## 4. Materials and Methods

### 4.1. Plant Material

Nine pea lines (*Pisum sativum* L.) developed within the breeding program of the Agricultural Institute Osijek, Croatia, were used in this study. The lines were created within the breeding cycle that started in 2014, and the selection criterion was seed yield. All nine breeding lines had shown good performance in preliminary evaluations and exhibited sufficient phenotypic variation to allow the assessment of differences in seed productivity and its components. The pedigree and selected phenotypic characteristics of the nine breeding lines are presented in Table 5. The lines differed in parental origin, leaf type, maturity, and seed color.

### 4.2. Experimental Site and Climatic Conditions

Field experiments were conducted at the Experimental Station of the Agricultural Institute Osijek, Croatia (45°32′25.8″ N, 18°44′12.0″ E) over the period 2020–2024. Sowing was carried out in March in each growing season, while harvesting took place in late July, when the seeds had reached the technological maturity stage. In July 2023, during the ripening stage, a hailstorm accompanied by strong winds caused lodging and physical damage to the plants and pods, which prevented reliable assessment of individual yield components. Consequently, the data from 2023 were not available for analysis, and the analyses were based on four growing seasons (2020, 2021, 2022 and 2024).

The area has a humid subtropical climate (Cfa) with warm summers and moderately cold winters. The average annual temperature (30-year average) is about 11.5 °C, while the annual precipitation usually ranges from 650 to 700 mm according to the Croatian Meteorological and Hydrological Service (CMHS). Weather conditions differed between years (Table 6). The growing seasons also differed in temperature and precipitation during critical phases of pea development over the four years. Throughout the growing season in 2020, conditions were favorable for the growth, development, and reproductive stage of pea. During the growing season in 2021, high temperatures and low precipitation were recorded during June during the grain filling phase, while 2022 was characterized by above average temperatures from May to July and low precipitation in July. High precipitation was recorded in May 2024, while in the same year, above-average temperatures were from May to July, and especially in July when they were extremely high.

The soil at the experimental site is classified as eutric cambisol (A–(B)v–C). Soil chemical analysis showed a neutral to slightly acidic soil reaction (pH(H_2_O) = 7.00, pH(KCl) = 6.41), a humus content of 2.01%, available phosphorus (AL-P_2_O_5_) of 39.70 mg 100 g^−1^ soil and available potassium (AL-K_2_O) of 37.70 mg 100 g^−1^ soil.

The experiment was conducted at the target breeding environment where the evaluated lines had originally been developed and selected. Evaluating the lines over multiple growing seasons at this location allowed their responses to interannual environmental variation to be assessed under the conditions of their target breeding environment. The study focused mainly on year to year responses of yield components rather than broad geographic adaptation, which made comparisons between growing seasons more direct.

### 4.3. Experimental Design and Data Collection

The experiment was set up as a randomized complete block design (RCBD) with three replications in each growing season. A total of nine genotypes were evaluated annually over four growing seasons, resulting in 108 experimental plots (4 years × 9 genotypes × 3 replications). Each experimental plot had dimensions of 5 × 1 m, with a row spacing of 14.5 cm and seeds sown at approximately 4 cm intervals within rows. Crop management and plant protection were conducted uniformly across all growing seasons. Prior to sowing, NPK fertilizer (7:20:30) was applied at a rate of 200 kg ha^−1^, providing 14 kg N, 40 kg P_2_O_5_ and 60 kg K_2_O ha^−1^. Weed control was performed when pea plants had developed three true leaf stages using a combination of bentazone and imazamox in split applications. The second application was made approximately 10 days after the first when field conditions permitted. For the control of pea weevil (*Bruchus pisorum*), esfenvalerate was applied at the beginning of flowering and lambda-cyhalothrin was applied at the end of flowering. Lambda-cyhalothrin was also applied as needed for aphid control. No fungicides were applied during the experimental period.

Within each experimental plot, ten plants were randomly selected from the center row to avoid boundary effects. Seed productivity and yield component traits were measured for each individual plant separately. The traits evaluated included seed productivity per plant (YL), number of pods per plant (P), number of seeds per pod (S/P), and thousand-seed weight (TSW). All statistical analyses and interpretations of traits were therefore performed at the level of individual plants, rather than using plot averages.

The resulting dataset had a hierarchical structure in which plants were nested within experimental plots. To account for this composition, a plot identification (PID) variable was created. The PID represented a unique combination of year, genotype, and replicate, corresponding to a single experimental plot within the experiment. Consequently, all plants originating from the same plot shared the same PID value.

### 4.4. Statistical Analysis

All statistical analyses were performed using Jamovi statistical software (version 2.6; The Jamovi Project, Sydney, Australia), using the GAMLj (version 3.6.1) module to fit linear mixed-effects models based on the lme4 (version 1.1-35.5) framework in R (version 4.4). Mixed models were fitted using restricted maximum likelihood estimation (REML), while significance testing of fixed effects was based on Satterthwaite’s approximation of degrees of freedom.

Model I. Baseline mixed effects model
  YL_ijkl_ = μ + G_i_ + Y_j_ + (G × Y)_ij_ + PID_k_ + ε_ijkl_
  where
YL_(ijkl)_ = observed seed productivity per plant (g);
μ = overall mean;
G_i_ = fixed effect of genotype;
Y_j_ = fixed effect of year;
(G × Y)_ij_ = genotype × year interaction;
PID_k_ = random intercept for experimental plot;
ε_ijkl_ = residual error.

Model II. Mixed-effects model for yield components and year interactions
  YL_ijkl_ = μ + G*i* + Y_j_ + P_ijkl_ + S_ijkl_ + T_ijkl_ + (Y ×P)_j_ + (Y × S)_j_ + (Y × T)_j_ + PID_k_ + ε_ijkl_
  where
YL_(ijkl)_ = observed seed productivity per plant (g);
G_i_ = fixed effect of genotype;
Y_j_ = fixed effect of year;
P_(ijkl)_ = number of pods per plant (fixed covariate);
S_(ijkl)_ = number of seeds per pod (fixed covariate);
T_(ijkl)_ = thousand-seed weight (g) (fixed covariate);
PID_k_ = random intercept for experimental plot;
ε_ijkl_ = residual error;
(Y × P)_j_ = interaction between year and number of pods per plant;
(Y × S)_j_ = interaction between year and number of seeds per pod;
(Y × T)_j_ = interaction between year and thousand-seed weight.

Plants were nested within experimental plots. PID was therefore included as a random intercept to account for differences between plots and the non-independence of plants from the same plot. Each PID represented one experimental plot defined by year, genotype, and replicate, and all plants sampled from the same plot had the same PID.

Model I was used to quantify the effects of genotype, year and genotype × year interaction on seed productivity. Model II extended the baseline model by incorporating yield components together with their interactions with year, allowing evaluation of the associations between reproductive traits and seed productivity and whether these relationships varied among growing seasons, providing insight into phenotypic plasticity. Model assumptions were assessed by examining residual diagnostic plots for both models, including Q–Q plots and residuals versus fitted values.

To facilitate comparison of predictors measured on different numerical scales, the continuous yield-component variables (number of pods per plant, number of seeds per pod, and thousand-seed weight) were standardized using z-score transformation, and standardized regression coefficients (β) were estimated from Model II. Model fit was assessed using the marginal and conditional R^2^ values, Akaike’s information criterion (AIC), and the Bayesian information criterion (BIC), as well as the intraclass correlation coefficient (ICC). Differences were considered statistically significant at *p* < 0.05.

## 5. Conclusions

The results of this study showed that variation in pea seed productivity was strongly associated with differences among growing seasons and the genotype × year interaction. Inclusion of yield components in the mixed-effects models improved the interpretation of seed productivity variation in pea. Pod number showed the strongest association with seed productivity, but its relationship with seed productivity varied among growing seasons. In contrast, thousand-seed weight showed a more stable relationship with seed productivity across years. The results suggest that changes in one yield component can be partially compensated by changes in another under changing environmental conditions. For breeding purposes, pod number may therefore be more useful as a selection trait when its stability across growing conditions is considered rather than its maximum expression, while thousand-seed weight may provide a more consistent selection criterion when an appropriate seed-size range is maintained. Further studies across additional target environments are needed to determine whether these relationships are consistent across environments and how contrasting plasticity of yield components can be incorporated into breeding selection.

## Figures and Tables

**Figure 1:**
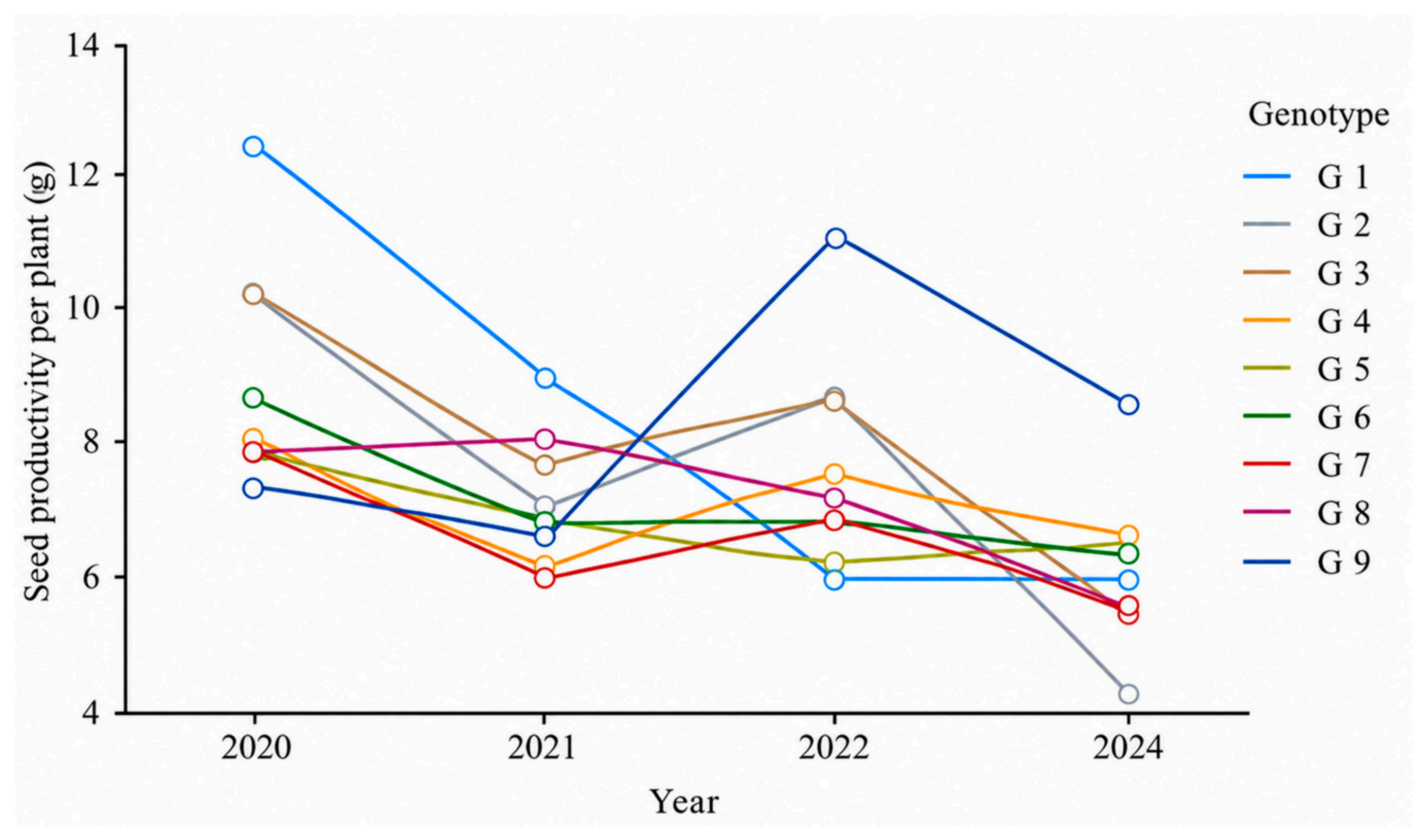
Genotype × year interaction for seed productivity per plant across four growing seasons.

**Figure 2:**
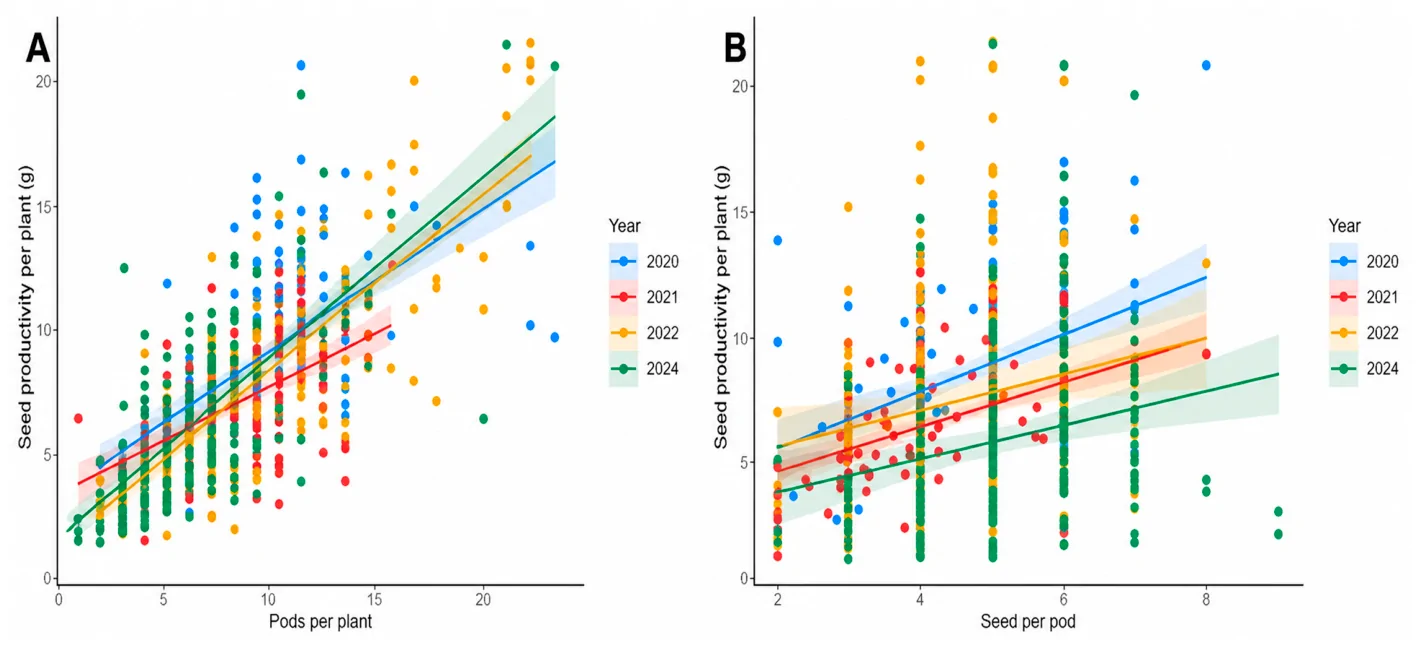
Relationships between seed productivity and (**A**) pods per plant and (**B**) seeds per pod across the experimental growing seasons. Points represent individual plant observations. Solid lines represent growing-season-specific linear regression trends, and shaded areas indicate 95% confidence intervals.

**Figure 3:**
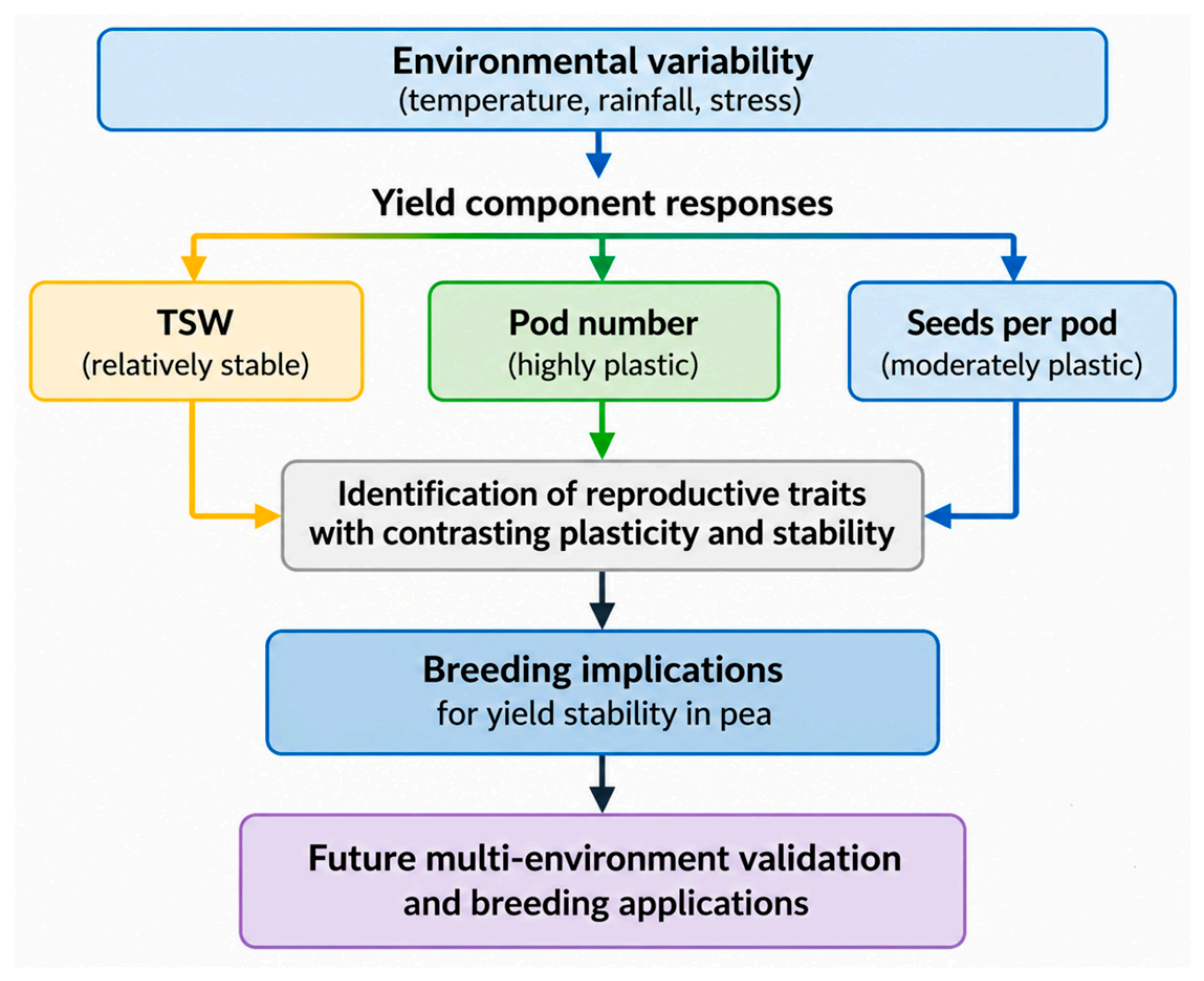
Conceptual breeding framework linking environmental variability, yield component plasticity and stability, and their implications for yield stability and future breeding applications. The framework is based on interannual variation observed at the experimental site in the present study.

**Table 1:** Descriptive statistics for seed productivity and yield components across growing seasons.

Year	*n*	Seed Productivity (Mean ± SD)	Pods Per Plant (Mean ± SD)	Thousand-Seed Weight (Mean ± SD)	Seeds Per Pod (Mean ± SD)
2020	270	8.86 ± 3.31	10.0 ± 3.35	186 ± 32.1	4.81 ± 1.14
2021	270	7.04 ± 2.36	9.07 ± 2.63	183 ± 34.3	4.53 ± 1.23
2022	270	7.60 ± 4.27	9.51 ± 4.72	182 ± 33.7	4.53 ± 1.12
2024	270	6.17 ± 3.58	6.98 ± 3.29	166 ± 46.8	5.22 ± 1.32

*n* = 270 individual plants per year (9 genotypes × 3 replications × 10 plants per plot).

**Table 2:** Linear mixed-effects model (Model I) for seed productivity across years and genotypes.

Source	Statistic	*p*-Value
Fixed effects		
Year	F = 15.19	<0.001
Genotype	F = 1.94	0.066
Genotype × Year	F = 2.57	0.001
Random effects		
PID variance	0.815	–
Residual variance	10.164	–
ICC (%)	7.4	–
Model fit		
Marginal R^2^	0.190	–
Conditional R^2^	0.250	–
AIC	3930	–
BIC	4106	–

**Table 3:** Results of the linear mixed-effects model (Model II) including yield components and year interactions.

Source	Statistic	*p*-Value
Fixed effects		
Year	F = 8.36	<0.001
Genotype	F = 3.49	0.001
Pods per plant	F = 1617.35	<0.001
Seeds per pod	F = 468.46	<0.001
Thousand-seed weight	F = 378.81	<0.001
Interactions		
Year × Pods per plant	F = 10.99	<0.001
Year × Seeds per pod	F = 8.40	<0.001
Year × Thousand-seed weight	F = 0.20	0.896
Random effects		
PID variance	0.065	–
Residual variance	2.251	–
ICC (%)	2.8	–
Model fit		
Marginal R^2^	0.820	–
Conditional R^2^	0.825	–
AIC	2897	–
BIC	3017	–

**Table 4:** Standardized effects of yield components on seed productivity per plant.

Yield Component	Standardized β	SE	*p*-Value
Pods per plant	2.743	0.061	<0.001
Thousand-seed weight	1.356	0.066	<0.001
Seeds per pod	1.255	0.061	<0.001

**Table 5:** Pedigree and selected phenotypic characteristics of the nine pea breeding lines.

Genotype	Pedigree	Leaf Type	Maturity	Seed Color
G-1	SANTANA × EIFFEL	Afila	Medium-early	Cream
G-2	EIFFEL × SOMERWOOD	Normal	Medium-early	Green
G-3	DOVE × FAKTOR	Afila	Medium	Cream
G-4	FAKTOR × AFILA PLUS	Afila	Medium	Green
G-5	DOVE × JEZERO	Afila	Medium-early	Yellow
G-6	ADEPT × ZEKON	Normal	Medium-late	Yellow
G-7	CORAL × SKINADO	Normal	Medium-late	Green
G-8	VALVERDE × SOLARA	Normal	Medium-late	Green
G-9	OSTEREICH-2 × SOLARA	Afila	Medium	Cream

**Table 6:** Monthly rainfall and mean air temperature recorded during the experimental growing seasons and corresponding long-term averages.

	Year	March	April	May	June	July
Rainfall (mm)	2020	37.4	20.7	53.3	73.5	40.0
	2021	34.4	60.7	58.9	18.4	96.7
	2022	6.4	35.0	66.0	77.2	19.2
	2024	20.1	15.7	131.8	74.2	47.0
	1995–2024	44.8	53.8	58.5	88.0	64.8
Temperature (°C)	2020	7.2	12.2	15.3	20.2	22.3
	2021	5.8	9.4	15.4	23.0	24.6
	2022	5.6	10.7	19.0	23.3	23.8
	2024	10.8	14.5	18.7	23.5	26.0
	1995–2024	6.4	11.1	16.1	19.1	21.1

Source: Croatian Meteorological and Hydrological Service, CMHS.

## Data Availability

The data presented in this study are available on request from corresponding author. The data are not publicly available due to ongoing research using a part of the dataset.

## Abbreviations

The following abbreviations are used in this manuscript:

AIC	Akaike Information Criterion
BIC	Bayesian Information Criterion
ICC	Intraclass Correlation Coefficient
PID	Plot Identification variable (unique experimental plot identifier)
RCBD	Randomized Complete Block Design
REML	Restricted Maximum Likelihood
TSW	Thousand-Seed Weight
